# Facile Solution Synthesis of Tungsten Trioxide Doped with Nanocrystalline Molybdenum Trioxide for Electrochromic Devices

**DOI:** 10.1038/s41598-017-13341-z

**Published:** 2017-10-16

**Authors:** Amirhossein Hasani, Quyet Van Le, Thang Phan Nguyen, Kyoung Soon Choi, Woonbae Sohn, Jang-Kyo Kim, Ho Won Jang, Soo Young Kim

**Affiliations:** 1 School of Chemical Engineering and Materials Science, Integrative research center for two-dimensional functional materials, Institute of Interdisciplinary Convergence Research, Chung-Ang University, 84 Heukseok-ro, Dongjak-gu, Seoul 06974 Republic of Korea; 2 Advanced Nano-Surface Research Group, Korea Basic Science Institute (KBSI), 169-148, Gwahak-ro, Yuseong-gu, Daejeon 34133 Republic of Korea; 3 Department of Materials Science and Engineering, Research Institute of Advanced Materials, Seoul National University, Seoul, 08826 Republic of Korea; 4 Department of Mechanical Engineering, The Hong Kong University of Science and Technology, Clear Water Bay, Kowloon, Hong Kong P.R. China

## Abstract

A facile, highly efficient approach to obtain molybdenum trioxide (MoO_3_)-doped tungsten trioxide (WO_3_) is reported. An annealing process was used to transform ammonium tetrathiotungstate [(NH_4_)_2_WS_4_] to WO_3_ in the presence of oxygen. Ammonium tetrathiomolybdate [(NH_4_)_2_MoS_4_] was used as a dopant to improve the film for use in an electrochromic (EC) cell. (NH_4_)_2_MoS_4_ at different concentrations (10, 20, 30, and 40 mM) was added to the (NH_4_)_2_WS_4_ precursor by sonication and the samples were annealed at 500 °C in air. Raman, X-ray diffraction, and X-ray photoelectron spectroscopy measurements confirmed that the (NH_4_)_2_WS_4_ precursor decomposed to WO_3_ and the (NH_4_)_2_MoS_4_–(NH_4_)_2_WS_4_ precursor was transformed to MoO_3_-doped WO_3_ after annealing at 500 °C. It is shown that the MoO_3_-doped WO_3_ film is more uniform and porous than pure WO_3_, confirming the doping quality and the privileges of the proposed method. The optimal MoO_3_-doped WO_3_ used as an EC layer exhibited a high coloration efficiency of 128.1 cm^2^/C, which is larger than that of pure WO_3_ (74.5 cm^2^/C). Therefore, MoO_3_-doped WO_3_ synthesized by the reported method is a promising candidate for high-efficiency and low-cost smart windows.

## Introduction

Electrochromic (EC) materials have attracted much attention owing to their potential applications in smart windows, antiglare mirrors, data storage devices, displays, sunroofs, and sunglasses. Various materials can be used as an EC layer, including inorganic metal oxides and organic conducting polymers^1–8^.

Conducting polymers provide benefits such as multiple colors, a fast switching time, and flexibility, but their disadvantages, including relatively nonuniform films, low material stability, and a limited range of colors severely limit their practical applications^9,10^. On the other hand, tungsten trioxide (WO_3_) is a well-known metal oxide owing to its excellent EC performance. WO_3_ with different structures has been prepared by techniques such as hydrothermal process, chemical vapor deposition, thermal evaporation, and sputtering^11–15^. However, these approaches have drawbacks that restrict the commercial application of WO_3_ EC films, including complicated preparation, high energy consumption, expensive equipment, or the use of toxic and dangerous reagents^16^.

Molybdenum oxide is one of the important semiconducting metal oxides and can be used in various applications, including photovoltaic cells, organic light-emitting diodes, gas sensors, hydrogen evolution systems, transistors, and EC devices^17–22^. However, the coloration efficiency of pure molybdenum oxide used in EC devices is not high. For example, Patil *et al*. found that MoO_3_ used as an EC layer had a coloration efficiency of 34 cm^2^/C^23^. A combination of tungsten and molybdenum oxide has been used in EC devices recently. For instance, Mahdavi *et al*. investigated the effect of molybdenum in a WO_3_ thin film prepared by RF magnetron sputtering and obtained a coloration efficiency of 42.5 cm^2^/C^24^. Kharade *et al*. synthesized MoO_3_ mixed with WO_3_ using a hybrid physicochemical method and achieved a high coloration efficiency of 121.56 cm^2^/C^25^. However, the complicated synthesis method with high cost is a drawback. Consequently, it is urgently necessary to develop immediate, effective, and facile methods to synthesize tungsten oxide films with enhanced EC performance.

In this study, we report a facile, low-cost method of producing EC thin films based on WO_3_ for smart window applications. In our previous work, we investigated the (NH_4_)_2_WS_4_ precursor annealed at 350 °C as a hole transport layer in an organic solar cell^26^. In this work, we investigate the use of different annealing temperatures to obtain WO_3_, and then we add the (NH_4_)_2_MoS_4_ precursor as a dopant to the (NH_4_)_2_WS_4_ precursor at different concentrations (10, 20, 30, and 40 mM) to obtain optimal EC films. A spin-coating method with an annealing process was applied to obtain EC films with excellent features such as high EC energy efficiency, high coloration efficiency, low cost, excellent chemical stability, fast switching speed, and good adhesion to the substrate.

## Results and Discussion

**Figure 1:**
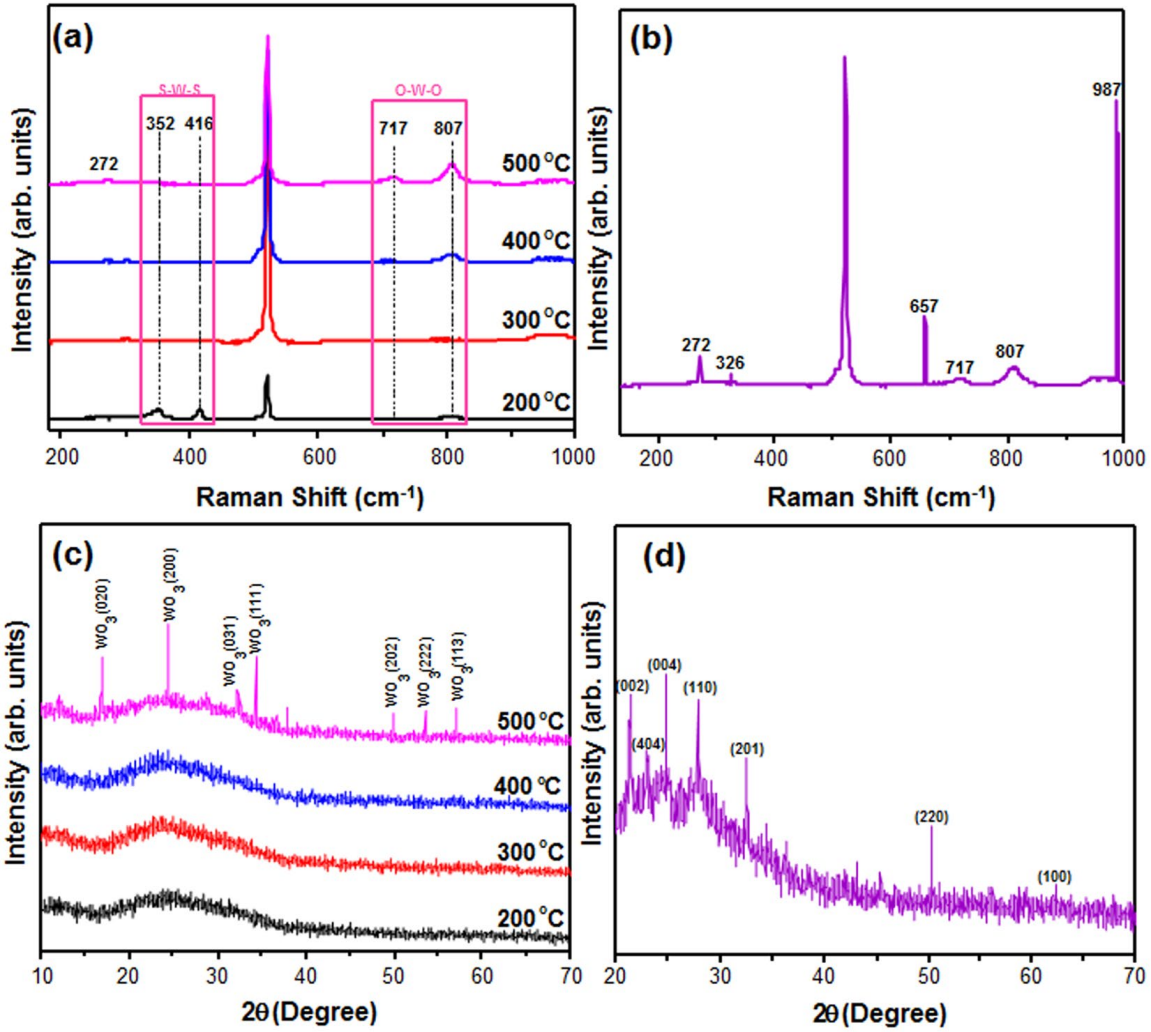
(**a**) Raman spectra of (NH_4_)_2_WS_4_ films annealed at different temperatures, (**b**) Raman spectra of (NH_4_)_2_WS_4_/(NH_4_)_2_MoS_4_ (30 mM) film annealed at 500 °C, (**c**) XRD pattern of (NH_4_)_2_WS_4_ film annealed at different temperatures, (**d**) XRD pattern of (NH_4_)_2_WS_4_/(NH_4_)_2_MoS_4_ (30 mM) film annealed at 500 °C.

The Raman spectra of the (NH_4_)_2_WS_4_ films annealed at different temperatures are shown in Fig. 1(a). The Raman peak of the S–W–S stretching mode is weakened for the amorphous phase as the temperature is increased, whereas the peaks corresponding to WO_3_ phases (O–W–O bending and stretching) are strengthened^27^. Therefore, the WO_3_ phases are completely decomposed after annealing at 500 °C, indicating a monoclinic crystal system. Figure 1(b) shows the Raman spectra of the MoO_3_-doped WO_3_ film. The peak at 675 cm^−1^ is ascribed to the coordinated oxygen in Mo crystal structure and stretching mode, which confirms that (NH_4_)_2_MoS_4_ was transformed to a MoO_3_ crystal, as indicated by the edge-shared oxygen^28^. Moreover, the Raman peaks at 272 cm^−1^ is assigned to O=Mo=O wagging modes^28^. Fig. 1(c) shows the XRD patterns of (NH_4_)_2_WS_4_ thin films annealed at different temperatures. The pristine WS_4_ shows broad WS_2_ peaks (2θ = 15–35°) related to weak crystallinity at annealing temperatures below 400 °C. The broad peak intensity decreases after annealing at 500 °C, and the peaks corresponding to WO_3_ structure are observed as well. These peaks are quite similar to those reported for monoclinic WO_3_^29^. Fig. 1(d) confirms the nanocrystallinity of the MoO_3_-doped WO_3_ films. The molybdenum is incorporated into the film, producing a new phase with orthorhombic crystal structure which is similar to the previously reported structures^24,30^. Moreover, the XRD results of the MoO_3_-doped WO_3_ did not show the peaks related to metallic Mo or MoO_3_, suggesting the well-diffusion of molybdenum atoms into WO_3_ crystal structure and substitution of Mo in W sites^24^.

**Figure 2:**
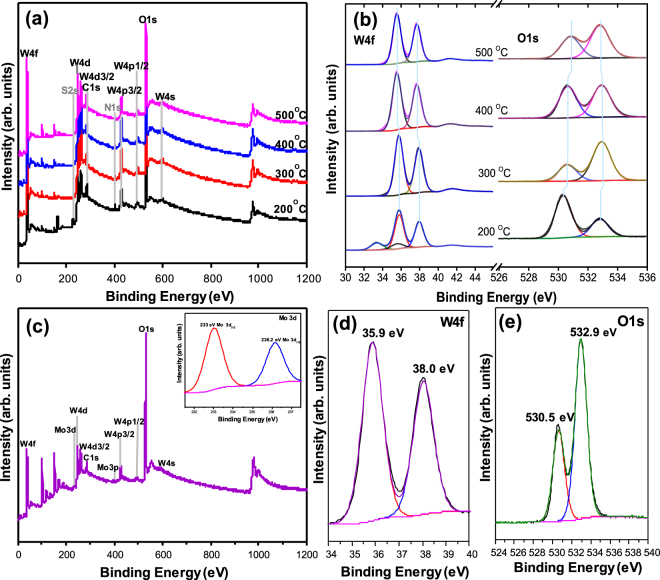
(**a**) XPS survey spectra of (NH_4_)_2_WS_4_ films annealed at different temperatures, (**b**) high-resolution W 4 f and O 1 s spectra of (NH_4_)_2_WS_4_ films annealed at different temperatures, (**c**) XPS survey spectra of (NH_4_)_2_WS_4_/(NH_4_)_2_MoS_4_ (30 mM) film annealed at 500 °C with high-resolution Mo 3d spectrum (inset), (**d**) W 4 f and (**e**) O 1 s spectra of (NH_4_)_2_WS_4_/(NH_4_)_2_MoS_4_ (30 mM) film annealed at 500 °C.

The XPS spectra of (NH_4_)_2_WS_4_ films annealed at different temperatures are shown in Fig. 2(a). As the temperature increases to 500 °C, the S 2 s and N 1 s peaks related to the (NH_4_)_2_WS_4_ precursor disappear, suggesting the complete decomposition of the (NH_4_)_2_WS_4_ precursor into WO_3_. WO_3_ appears owing to the presence of O_2_ in the air^26^. High-resolution views of the W 4 f and O 1 s peaks are shown in Fig. 2(b). The peak appears at 33.5 eV for the (NH_4_)_2_WS_4_ film annealed at 200 °C, and the peaks at 35.4, 36.3, and 38 eV are ascribed to W^4+^, W^5+^, and W^6+^ ^26^. The peak of W^5+^ is related to oxygen vacancy^31^. As the annealing temperature increases, the peak located at 33.5 eV vanish, confirming the transformation of WS_2_ to WO_3_. The observed shifts of the peaks in the O 1 s and W 4 f spectra toward lower binding energy can be attributed to the emission of photoelectrons from the higher to lower oxidation states of W^32^. For the XPS data in the O 1 s region, the peak density related to the oxide phase near 531 eV increases as the annealing temperature increases beyond 300 °C (Fig. 2(b))^33^. The XPS survey scan of the MoO_3_-doped WO_3_ film (Fig. 2(c)) shows additional peaks that are related to Mo 3d and Mo 3p. The XPS peak positions of Mo 3d_3/2_ and Mo 3d_1/2_ are 233 and 236.2 eV, respectively (inset of Fig. 2(c)), which are attributed to pair of orbital spinning of MoO_3_^34^. The two observed peaks of W 4f_7/2_ and W 4f_5/2_ appear at 35.9 and 38 eV, respectively (Fig. 2(d)). Figure 2(e) shows the high-resolution XPS O 1 s spectrum, in which the oxygen O 1 s peaks are observed at 530.5 and 539.2 eV. The spectra indicate the presence of W, Mo, and O in the as-prepared MoO_3_-doped WO_3_ EC film with oxidation states of +6, +6, and −2, respectively^25^. All of XPS results and observed peaks are confirmed by previously reported works^25–35^.

**Figure 3:**
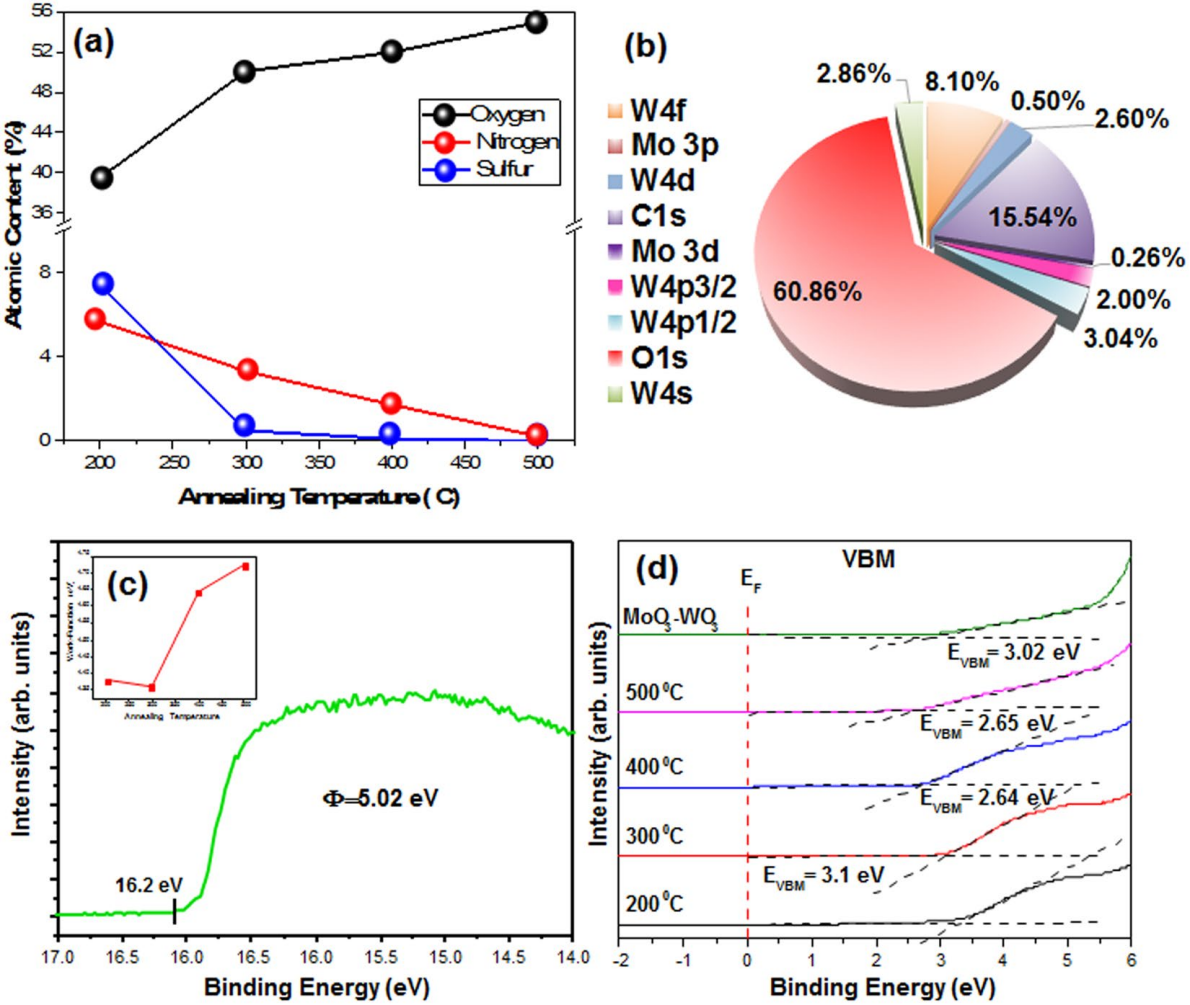
(**a**) Atomic ratios of (NH_4_)_2_WS_4_ films annealed at different temperatures, (**b**) atomic content of (NH_4_)_2_WS_4_/(NH_4_)_2_MoS_4_ (30 mM) film annealed at 500 °C, (**c**) UPS spectra of (NH_4_)_2_WS_4_/(NH_4_)_2_MoS_4_ (30 mM) film annealed at 500 °C and (inset) work function of (NH_4_)_2_WS_4_ films annealed at different temperatures, (**d**) ultraviolet photoelectron spectra of (NH_4_)_2_WS_4_ films annealed at different temperatures and (NH_4_)_2_WS_4_/(NH_4_)_2_MoS_4_ (30 mM) film annealed at 500 °C.

The atomic ratios of (NH_4_)_2_WS_4_ films annealed at different temperatures are shown in Fig. 3(a). Those of sulfur (S 2 s) and nitrogen (N 1 s) approach zero as the annealing temperature is increased to 400 °C. The pie chart in Fig. 3(b) indicates that the oxygen content of the MoO_3_-doped WO_3_ is higher than that of other atoms owing to the annealing process and the presence of oxygen in both the MoO_3_ and WO_3_ structures. It is calculated that the level of doping was 0.76% (30 mM (NH_4_)_2_MoS_4_ into (NH_4_)_2_WS_4_ precursor). These results not only support the formation of an oxide surface layer on the sulfide (NH_4_)_2_WS_4_ backbone, but also represent the functionalization of MoO_3_ on the WO_3_ structure. As shown in the inset of Fig. 3(c), the work function of the pure WO_3_ thin film increases as the annealing temperature increased to 500 °C. The work function of WO_3_ at 500 °C is 4.71 eV (inset of Fig. 3(c)). The increase of work function is attributed to the formation of WO_3_^26^. In addition, the green curve shown in Fig. 3(c) indicates that the work function of MoO_3_-doped WO_3_ at 500 °C is 5.02 eV, which is higher than that pure WO_3_. These data suggest better performance of electrochromic in MoO_3_-doped WO_3_ device by facilitating charge transfer. Figure 3(d) shows the valance band maxima (VBMs) of the WO_3_ and MoO_3_-doped WO_3_, which decrease with increasing annealing temperature. However, MoO_3_ doping method increases the VBM owing to changes in the O 1 s states and promoting the transition of intervalance within metal ions^34,36^. These data suggest the enhanced transition of electron in MoO_3_-doped WO_3_ film, resulting in increased coloration efficiency.

**Figure 4:**
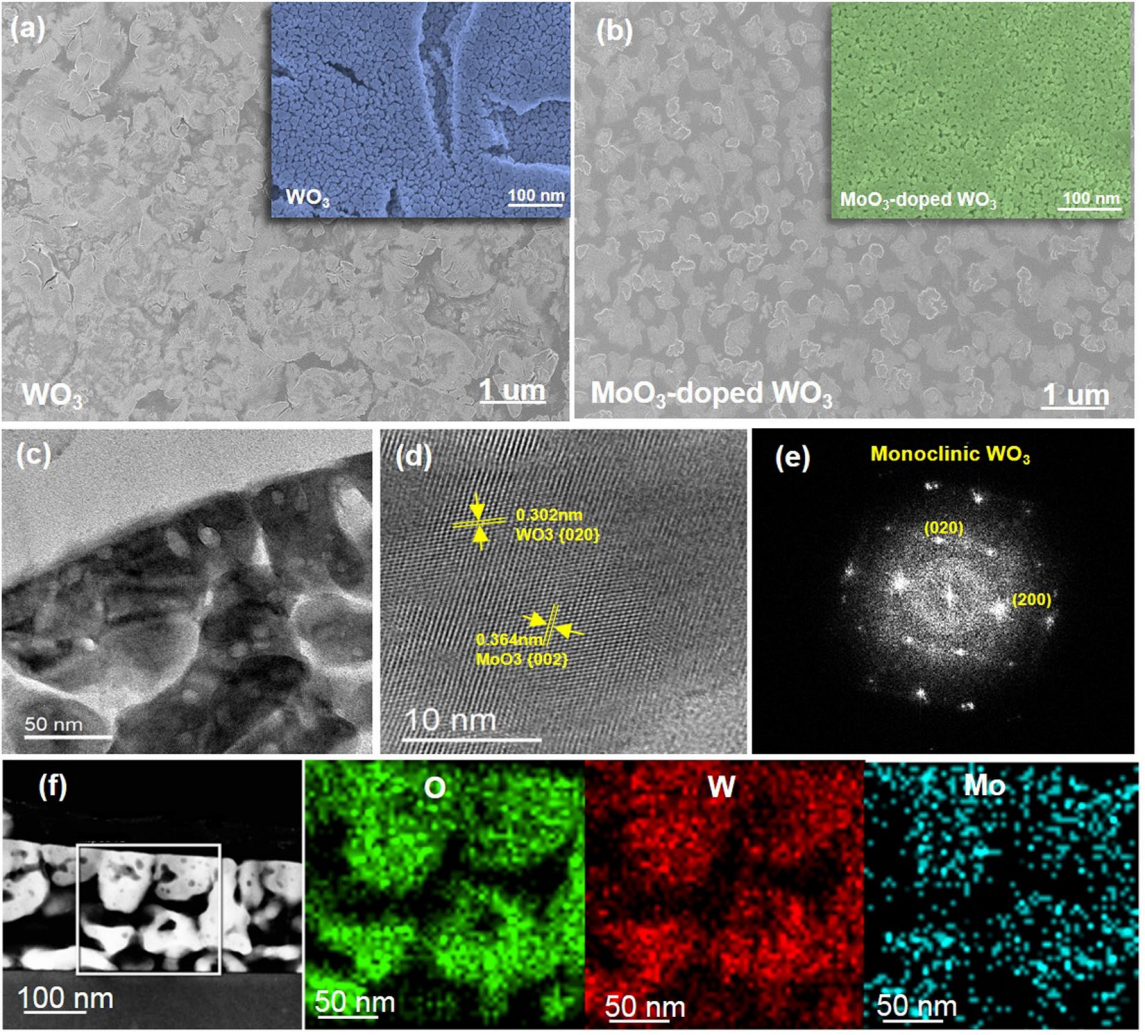
FESEM images of (**a**) (NH_4_)_2_WS_4_ film and (**b**) (NH_4_)_2_WS_4_/(NH4)_2_MoS_4_ (30 mM) film annealed at 500 °C, (**c**) TEM image of MoO_3_-doped WO_3_, (**d**) HRTEM image of MoO_3_-doped WO_3_, (**e**) electron diffraction and (**f**) STEM images and the corresponding STEM–EDX elemental maps of MoO_3_-doped WO_3_.

Figure 4 shows FESEM and HRTEM images of the WO_3_ and MoO_3_-doped WO_3_. Both samples have porous and compact surfaces. The observed cracks are attributed to the annealing process (Fig. 4(a) and (b)). Moreover, the MoO_3_-doped WO_3_ exhibits a more uniform than pure WO_3_ (Fig. 4(c)). In addition, porosity measurement was carried out by using MATLAB software whose method was previously reported^37^. In order to measure the porosity, the FESEM images of WO_3_ and MoO_3_-doped WO_3_ were converted to binary image (see Figure S1) and then the percentage of porosity was calculated by following formula:

1 $$P=(1-\frac{{\rm{n}}}{{\rm{N}}})\times 100$$

where P is the porosity percent, n is the number of white pixels, and N is the total number of white and black pixels. The percentage of porosities were obtained 58.2 and 75.6% in WO_3_ and MoO_3_-doped WO_3_ film, respectively. The lattice fringes in the HRTEM images demonstrate that the WO_3_ films are crystallized, confirming the XRD results. In addition, the estimated lattice spacings of 0.302 and 0.364 nm are assigned to the monoclinic d-spacing of the (020) plane of WO_3_ and the orthorhombic (002) plane of MoO_3_ (Fig. 4(d) and (e)). Figure 4(f) shows the elemental distributions of W, O, and Mo for the MoO_3_-doped WO_3_ thin film. MoO_3_ atoms not only grew on the WO_3_ surface, but are also diffused into the film. These data indicate that the method has great potential for efficient doping.

**Figure 5:**
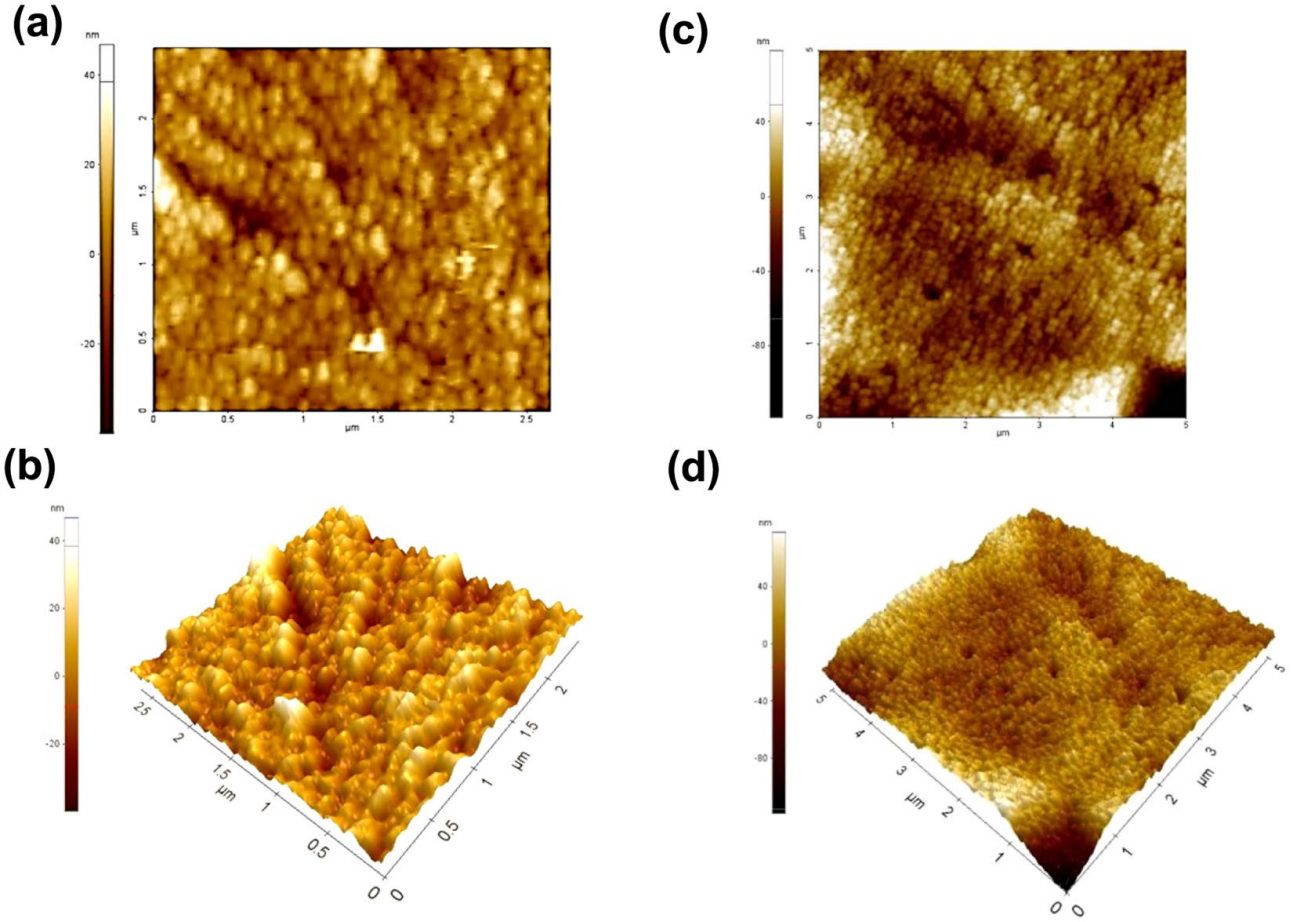
(**a**) 2D and (**b**) 3D AFM images of (NH_4_)_2_WS_4_ film annealed at 500 °C, (**c**) 2D and (**d**) 3D AFM images of (NH_4_)_2_WS_4_/(NH_4_)_2_MoS_4_ (30 mM) film annealed at 500 °C.

According to the AFM images (Fig. 5), the measured roughness of the WO_3_ and MoO_3_-doped WO_3_ is 6.5 and 2.4 nm, respectively. Therefore, the uniformity of film is increased without aggregation caused by doping. The AFM images confirm that the porosity of the film is greater after doping. The higher porosity is expected to improve the EC performance by enhancing the diffusion constant of the intercalating ions through the pore interface^38^. In addition, higher roughness and porosity of film can improve the distribution of electrical field during redox process resulting in enhanced electron transfer and ion-insertion, leading to high EC performance^24,34,39,40^.

**Figure 6:**
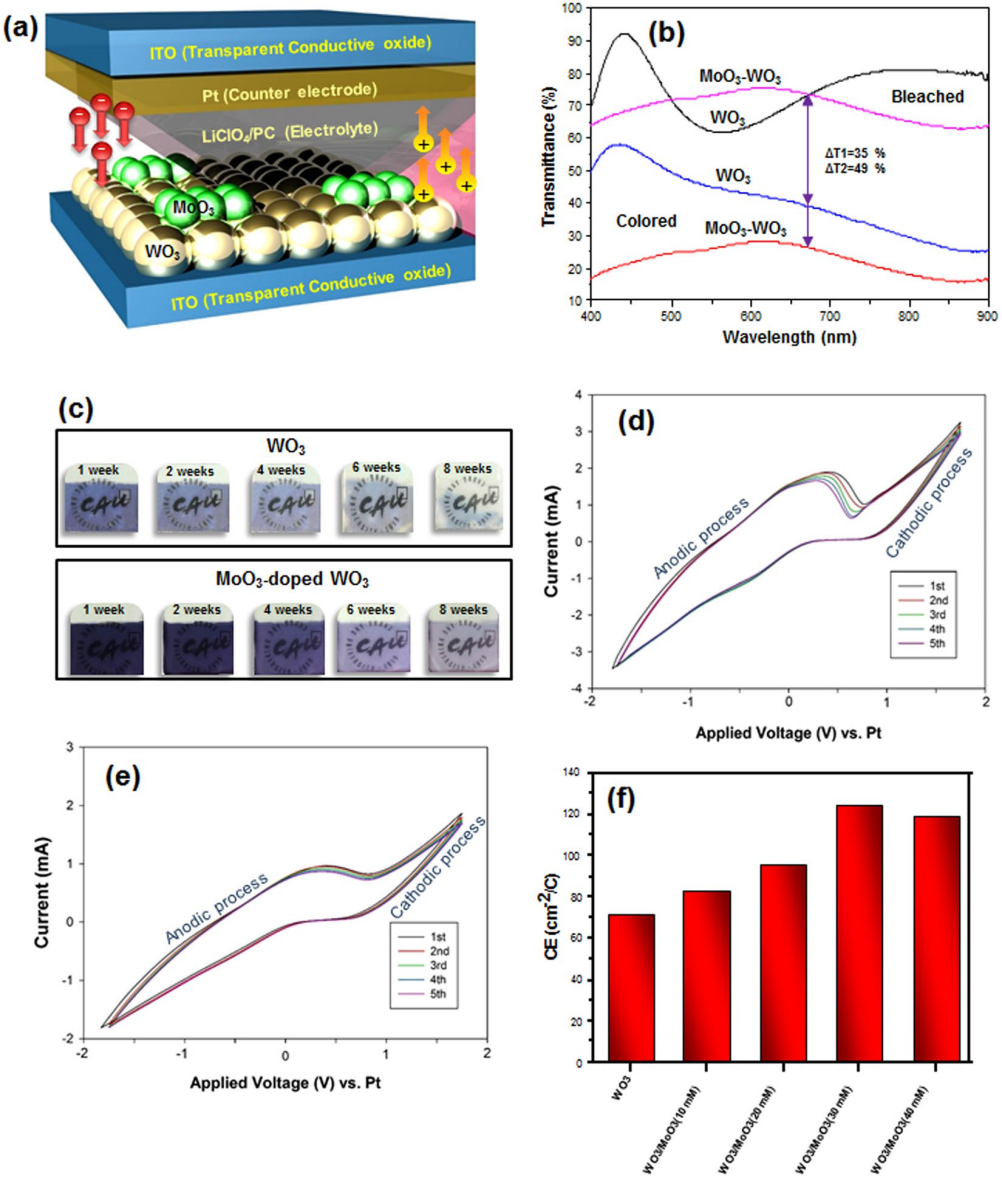
(**a**) Structure of as-prepared EC cell, (**b**) transmission spectra of WO_3_ and MoO_3_-doped WO_3_, (**c**) memory behavior of WO_3_ and MoO_3_-doped WO_3_ films during 8 weeks, CV curves of (**d**) WO_3_ and (**e**) MoO_3_-doped WO_3_ EC films and (**f**) coloration efficiency of the different EC films.

Figure 6(a) shows the configuration of the EC cell. The transmittance spectra (wavelength 400–900 nm) of WO_3_ and MoO_3_-doped WO_3_ in the colored and bleached states were measured (Fig. 6(b)). The color was changed to dark blue when a DC voltage of −2.5 V was applied across the ITO. After the voltage was changed to +2.5 V, the EC cell returned to the transparent state. The mechanism is thought to be the oxidation and reduction process. Li^+^ ions are inserted into the EC film, leading to reduction of W^6+^ to W^5+^ and the increase in the cathodic current change the color of the film. The change from the colored state to the transparent state is ascribed to oxidation (W^5+^ to W^6+^) due to the changing in redox state of the tungsten ions and the number of electrons (charge) inserted into EC film^24^. In the doped EC film, Mo is involved in the reduction/oxidation process (Mo^6+^ to Mo^5+^ and Mo^5+^ to W^6+^), which causes to the enhanced transition of intervalency and electron transition within ions^34^. Optical modulation is one of the most important parameters in EC devices and can be defined as ΔT = T_b_ − T_c_, where T_b_ and T_c_ are the transmittance in the bleached and colored states, respectively, at a particular wavelength^41^. The difference in transmission (ΔT at 675 nm) between the bleached and colored states in WO_3_ and MoO_3_-doped WO_3_ was enhanced from ΔT_1_ = 35% to ΔT_2_ = 49%. This improvement is attributed to a change in the crystal structure of WO_3_ caused by substitution of Mo at W sites and charge transfer between the Mo^5+^ and W^6+^ sites^24,42^. Fig. 6(c) shows the stability of the WO_3_ and MoO_3_-doped WO_3_ in the colored state for several weeks after the voltage is removed. MoO_3_-doped WO_3_ exhibited better memory behaviour in air, in which it relatively retained the colored state very well even after 4 weeks. On the other hand, some parts of the pure WO_3_ EC film became transparent as time passed. Therefore, the memory behaviour of MoO_3_-doped WO_3_ is better than that of pure WO_3_ because of the increase in the diffusion coefficient (D) of Li^+^ ions in MoO_3_-doped WO_3_ during the intercalation process^24^. Diffusion coefficient during intercalation process can be calculated by Randles−Sevcik equation^24,43^:

2 $${{\rm{D}}}^{1/2}={{\rm{i}}}_{{\rm{p}}}/[(2.72\times {10}^{5}){{\rm{n}}}^{3/2}{{\rm{AC}}}_{{\rm{0}}}{{\rm{\pi }}r}^{{\rm{2}}}{{\rm{v}}}^{1/2}]$$

where i_p_ is the anodic peak current density, n is the number of electrons transferred during redox process, C_0_ is the concentration of active ions in the electrolyte, ν is the scan rate, and A is the area of the EC film^24^. The diffusion coefficient was obtained 1.23 × 10^−11^ and 9.42 × 10^−11^ for the WO_3_ and MoO_3_-doped WO_3_ EC film, respectively. Figure 6(d) and (e) shows the current-voltage (CV) curves of pure WO_3_ and MoO_3_-doped WO_3_ thin films, which were measured in a 1 M aqueous solution at a scan rate of 50 mV/s. In the cathodic process, the current of pure WO_3_ is higher than that of the MoO_3_-doped WO_3_ thin film. The MoO_3_-doped WO_3_ has a higher conductivity than pure WO_3_ because more defect states are created owing to integration of the two metal oxides, decreasing the energy required to extract the intercalated Li^+^ ions after MoO_3_ doping^24,25^. In addition, the cycling stability of both thin films after 300 cycle steps revealed that the current in the MoO_3_-doped WO_3_ did not change and remained constant compared to that of the pure WO_3_ EC film. Moreover, the CV curves not only indicate a well-crystallized WO_3_ structure in both films, but also confirm the XRD and TEM results.

The coloration efficiency (CE), which is an important parameter for EC devices, was calculated as follows^23,24,30^:

3 $${\rm{CE}}=\frac{{\rm{\Delta }}\mathrm{OD}}{{\rm{Q}}}$$

4 $${\rm{\Delta }}\mathrm{OD}=\,\mathrm{log}[\frac{{{\rm{T}}}_{{\rm{b}}}}{{{\rm{T}}}_{{\rm{c}}}}]$$

where ΔOD is the change in optical density, Q is the charge density, T_b_ is the transmittance of the film in the bleached state, and T_c_ is the transmittance of the film in the colored state^23,24,41^. The color efficiency of various EC layers and various MoO_3_ concentrations (λ = 675 nm) is presented in Fig. 6(f). The CE values of the MoO_3_-doped WO_3_ are higher than that of pure WO_3_ (74.5 cm^2^/C). To determine the optimal concentration of MoO_3_ in WO_3_, the CE was measured for different MoO_3_ concentrations (10, 20, 30, and 40 mM) in WO_3_. As shown in Fig. 6(f), a much higher CE (128.1 cm^2^/C) was obtained for the MoO_3_-doped WO_3_ with a MoO_3_ concentration of 30 mM. This result indicates that the optimal amount of molybdenum has a crucial role in obtaining high efficiency. Furthermore, the response times of the colored and bleached states for MoO_3_-doped WO_3_ are found to be 3.6 and 4.5 s, respectively, whereas the response times of the colored and bleached states for pure as-prepared WO_3_ are 8 and 9.5 s, respectively. Enhanced EC properties is caused by improvement in extra electron intervalance transfer W^6+^ and Mo^6+^ active sites. In addition, molybdenum after intercalation of Li^+^ ions are more near to the sensitivity of human’s vision. The disorder could be increased by random distribution of molybdenum, resulting in the betterment EC properties^25^. Therefore, the performance was improved by MoO_3_ doping. Table 1 compares our results with the reported values through various materials and methods.

**Table 1:** Comparison of our work with previously published papers.

Material	Method	Switching time (t_c_/t_b_)	ΔT (%)	Coloration efficiency (cm^2^/C)	Ref.
Mo-doped WO_3_	RF magnetron sputtering	—	44.3	42.5	24
MoO_3_/WO_3_	Hybrid physicochemical synthesis	4.1 s/3.4 s	~50	121.56	25
Nanoparticulate WO_3_	Electrodeposition	3.7 s/5.2 s	88.51	137	35
NiO/WO_3_	DC magnetron sputtering	10 s/20 s	55	87	40
Ti-doped WO_3_	Sol–gel spin-coating	—	47.5	—	44
PANI/WO_3_	Electropolymerization	9.9 s/13.6 s	37.4	98.4	45
WO_x_ nanorods	Low-temperature ozone exposure	11.8 s/20.1 s	57	33.3	46
WONWS-RGO	Solvothermal	1.5 s/1.2 s	—	116.7	47
MoO_3_-doped WO_3_	Solution and annealing process	3.6 s/4.5 s	49	128.1	Our work

## Conclusions

WO_3_ doped with MoO_3_ was prepared by a facile and low-cost method involving solution and annealing processes. The results indicated that the (NH_4_)_2_MoS_4_/(NH_4_)_2_WS_4_ precursor decomposed to MoO_3_-doped WO_3_ when the film was annealed at 500 °C in air. The N 1 s and S 2 s emission in the XPS spectrum of the (NH_4_)_2_WS_4_ precursor annealed at 500 °C, as well as weakening of the S–W–S bond, increased O–W–O bond peaks in the Raman spectra, and the appearance of peaks in the XRD spectra, suggested full decomposition to monoclinic crystalline WO_3_. In addition, (NH_4_)_2_MoS_4_ was added to the (NH_4_)_2_WS_4_ precursor at various concentrations as a dopant, and the resulting films were then annealed at 500 °C to transform them to MoO_3_-doped WO_3_. The XRD and Raman spectroscopy results confirmed the decomposition to nanocrystalline MoO_3_-doped WO_3_. Moreover, the morphology of the as-prepared films was observed using FESEM and AFM, which showed that the MoO_3_-doped WO_3_ was more uniform and porous than pure WO_3_, suggesting high EC performance. Furthermore, the high doping capability with good distribution of MoO_3_ into WO_3_ was confirmed by HRTEM images. As a result, enhanced EC performance was obtained when the MoO_3_-doped (30 mM) WO_3_ was used as an EC layer. The coloration efficiency was high (CE = 128.1 cm^2^/C), and the response time was rapid (t_c_ = 3 s, t_b_ = 4.5 s). These values are much higher than those of pure WO_3_ (CE = 74.5 cm^2^/C, t_c_ = 8 s, t_b_ = 9.5 s). In conclusion, the MoO_3_-doped WO_3_ prepared by the annealing–solution process is a remarkable candidate for use in high-efficiency, low-cost smart windows that can be efficiently commercialized.

## Method

### Preparation of thin film of MoO_3_-doped WO_3_

**Figure 7:**
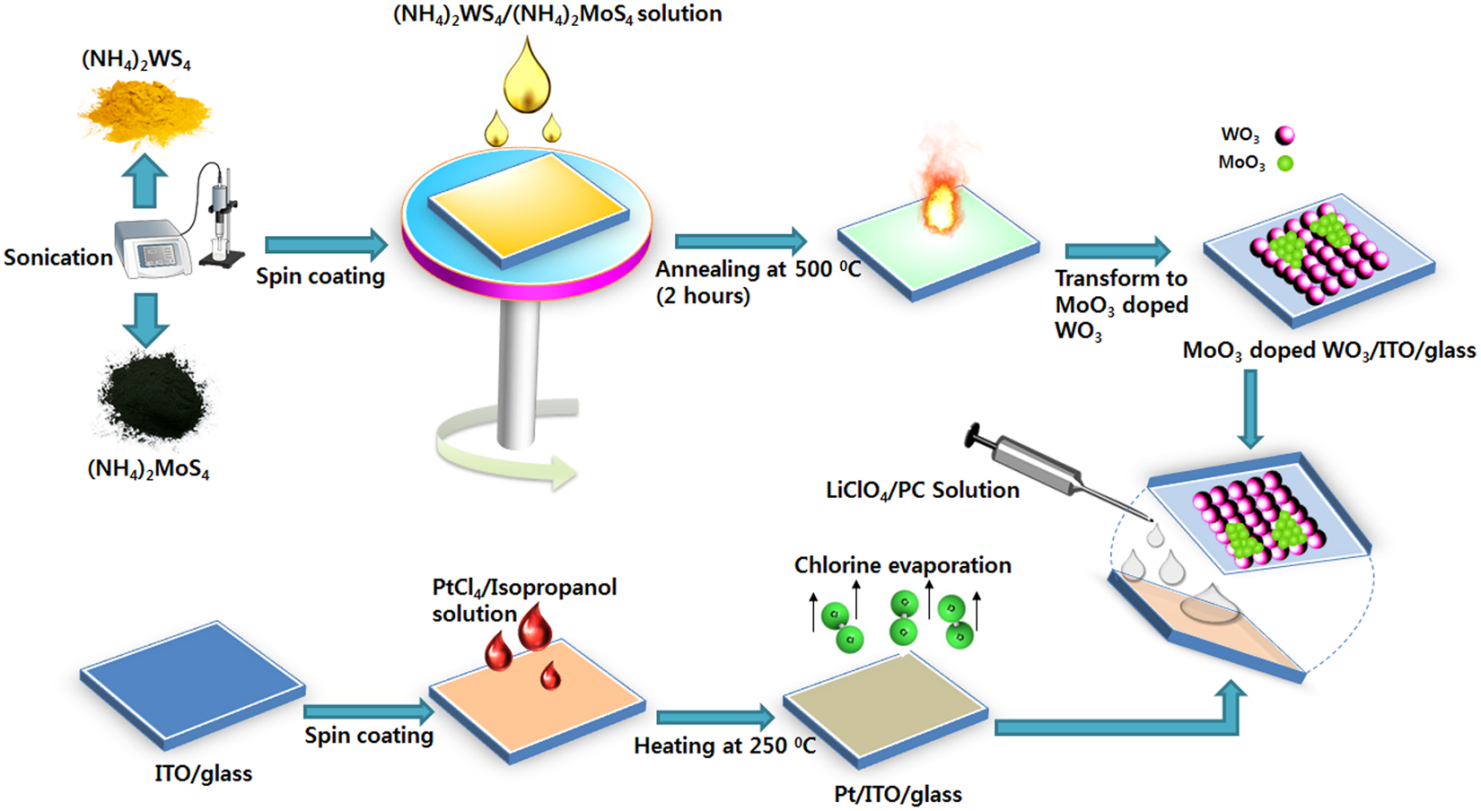
Synthesis of EC materials and fabrication of EC cell.

Figure 7 illustrates the synthesis of the WO_3_ and MoO_3_-doped WO_3_ thin films and fabrication of the EC cell. Indium tin oxide (ITO) substrates were ultrasonically cleaned sequentially with DI water, isopropanol, and acetone, and then dried; they were then treated by ultraviolet ozone for 20 min and maintained there until the start of the spin-coating process. After drying, PtCl_4_ dispersed in isopropanol was coated on one piece of the ITO conductive glass by spin-coating, and the ITO was dried on a hotplate at 250 °C to evaporate the solvent and chlorine; this sample was used as a counter electrode. Next, (NH_4_)_2_WS_4_ (200 mg) was dissolved in 1 ml of N,N-dimethylformamide, resulting in the formation of a yellowish tungsten sol. Then, a homogenous thin film was prepared by spin-coating the as-prepared solution onto the ITO substrate at 4000 rpm for 60 s. After drying in air, the coated substrates were annealed in a furnace at different temperatures (200, 300, 400, and 500 °C) for 2 h. A transparent, colorless WO_3_ thin film was obtained at 500 °C. To form the MoO_3_-doped WO_3_ thin films, (NH_4_)_2_MoS_4_ was added to the (NH_4_)_2_WS_4_ solution separately (at different concentrations, 10, 20, 30, and 40 mM, to determine the optimal amount of doping) to form a homogenous solution. Then, this procedure was repeated to synthesize MoO_3_-doped WO_3_ thin films at 500 °C.

### Fabrication of the EC cell

The EC device structure for the MoO_3_-doped WO_3_ thin films was glass/ITO/MoO_3_-doped WO_3_/LiClO_4_ + propylene carbonate (PC)/Pt/ITO/glass. The ITO substrate coated with the MoO_3_-doped WO_3_ thin film acts as a working electrode, and the Pt/ITO-coated conducting glass substrate acts as a counter electrode; the electrodes are assembled to fabricate a sandwich-type EC device. The liquid electrolyte, 1 M lithium perchlorate (LiClO_4_)/PC, was injected into the device through a small hole, which was then sealed with Resibond epoxy glue.

### Characterizations

X-ray photoelectron spectroscopy (XPS) was performed using an ESCA-3000 (VG Scientific Ltd., England) instrument analyzer under a vacuum better than 1 × 10^−5^ mbar using Mg Kα radiation (1250 eV) and a constant pass energy of 50 eV. The composition of the thin film samples was determined by X-ray diffraction (XRD) analysis (Bruker AXS Model D8 Advance X-ray diffractometer) with a Cu Kα target having a wavelength 0.1542 nm. Raman spectra (LabRAM HR, Horiba Jobin Yvon, Japan) were obtained at an excitation wavelength of 514 nm. Field-emission scanning electron microscopy (FESEM, Zeiss 300 VP) images were taken at an acceleration voltage of 50 kV. Transmission electron microscopy (TEM) was performed with a JEOL-2100F (Japan) instrument. Contact-mode atomic force microscopy (AFM, XE-100/PSIA) was used to determine the roughness and porosity of the thin films. Cyclic voltammetry (CV) and electrochemical measurements were performed in a quartz electrochemical cell connected to a potentiostat (Ivium 5612, Netherlands). WO_3_ or MoO_3_-doped WO_3_ was used as the working electrode, and a Ag/AgCl electrode and platinum (Pt) wire were applied as the reference and counter electrodes, respectively. The transmittance spectra were measured by a UV–vis spectrophotometer (V-670). The coloration/bleaching switching characteristics of the EC films were recorded as the changes in the transmittance at a wavelength of 675 nm under alternating application of a potential of ±2.5 V for 60 s for each state. For the XPS, high-resolution TEM (HRTEM), FESEM, XRD, Raman, and AFM measurements of the MoO_3_-doped WO_3_, samples fabricated using 30 mM of (NH_4_)_2_MoS_4_ in the (NH_4_)_2_WS_4_ precursor were used.

## Electronic supplementary material

Supplementary information
