# Supplementary material for: Facile Solution Synthesis of Tungsten Trioxide Doped with Nanocrystalline Molybdenum Trioxide for Electrochromic Devices
Source: Sci Rep. 2017 Oct 16;7:13258. doi: 10.1038/s41598-017-13341-z (PMC5643310; doi:10.1038/s41598-017-13341-z)

Supplementary Information

Facile Solution Synthesis of Tungsten Trioxide Doped with Nanocrystalline Molybdenum Trioxide for Electrochromic Devices

Amirhossein Hasani,[‡ a] Quyet Van Le,[‡ a] Thang Phan Nguyen,[a] Kyoung Soon Choi,[b] Woonbae Sohn,[c] Jang-Kyo Kim,[d] Ho Won Jang,[*c] Soo Young Kim[* a]

(a) School of Chemical Engineering and Materials Science, Integrative research center for two-dimensional functional materials, Institute of Interdisciplinary Convergence Research, Chung-Ang University, 84 Heukseok-ro, Dongjak-gu, Seoul 06974, Republic of Korea

(b) Advanced Nano-Surface Research Group, Korea Basic Science Institute (KBSI), 169-148, Gwahak-ro, Yuseong-gu, Daejeon 34133, Republic of Korea

(c) Department of Materials Science and Engineering, Research Institute of Advanced Materials, Seoul National University, Seoul 08826, Republic of Korea

(d) Department of Mechanical Engineering, The Hong Kong University of Science and Technology, Clear Water Bay, Kowloon, Hong Kong (P.R. China)

Corresponding authors: *Soo Young Kim, Tel.: +82-2-820-5875; Fax: +82-2-824-3495; E-mail: sooyoungkim@cau.ac.kr, *Ho Won Jang, Tel.: +82-2-880-1720; Fax: +82-2-885-9671; E-mail: hwjang@snu.ac.kr

Figure S1. (a) FESEM image of WO_3 and (b) MoO_3-doped WO_3 film, (c) binary image of WO_3 and (d) MoO_3-doped WO_3 film.

(a)

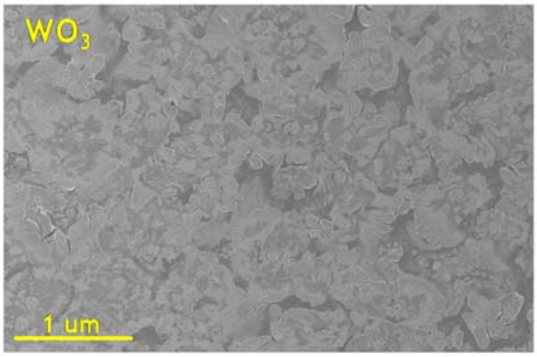

(b)

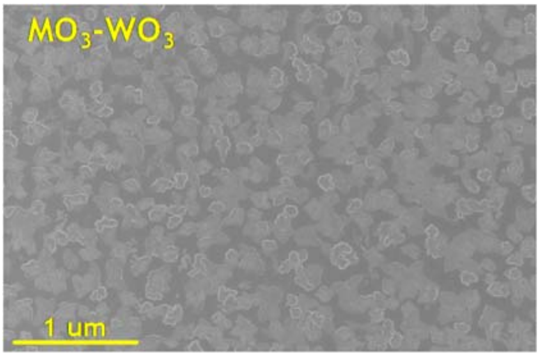

(c)

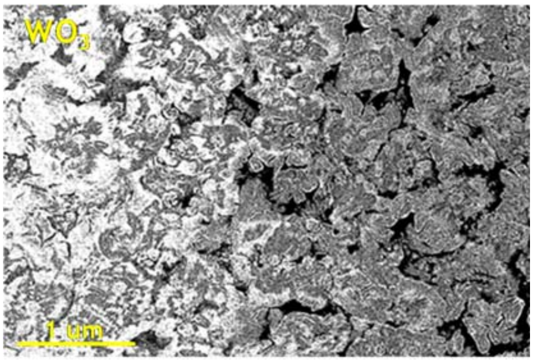

(d)

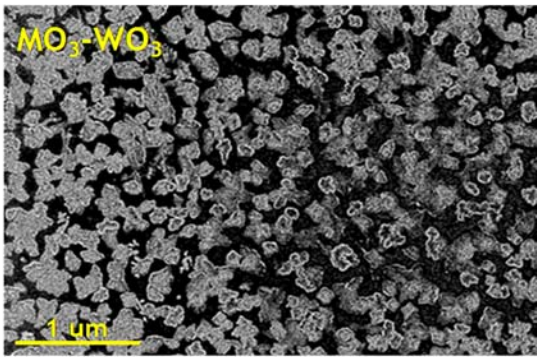

Supplement: Supplementary file 1 — Supplementary information [file 41598_2017_13341_MOESM1_ESM.pdf]
